# Unraveling the Mystery of Cold Stress-Induced Myocardial Injury

**DOI:** 10.3389/fphys.2020.580811

**Published:** 2020-11-05

**Authors:** Xue Kong, Haitao Liu, Xiaole He, Yang Sun, Wei Ge

**Affiliations:** Department of General Practice, Xijing Hospital, Fourth Military Medical University, Xi’an, China

**Keywords:** cold stress, hypothermia, myocardial injury, cardiac function, mechanism, molecular pathways

## Abstract

Exposure to low ambient temperature imposes great challenge to human health. Epidemiological evidence has noted significantly elevated emergency admission and mortality rate in cold climate in many regions, in particular, adverse events in cardiovascular system. Cold stress is becoming one of the important risk factors for cardiovascular death. Through recent advance in echocardiography and myocardial histological techniques, both clinical and experimental experiments have unveiled that cold stress triggers a variety of pathological and pathophysiological injuries, including ventricular wall thickening, cardiac hypertrophy, elevated blood pressure, decreased cardiac function, and myocardial interstitial fibrosis. In order to examine the potential mechanism of action behind cold stress-induced cardiovascular anomalies, ample biochemical and molecular biological experiments have been conducted to denote a role for mitochondrial injury, intracellular Ca^2+^ dysregulation, generation of reactive oxygen species (ROS) and other superoxide, altered gene and protein profiles for apoptosis and autophagy, and increased adrenergic receptor sensitivity in cold stress-induced cardiovascular anomalies. These findings suggest that cold stress may damage the myocardium through mitochondrial injury, apoptosis, autophagy, metabolism, oxidative stress, and neuroendocrine pathways. Although the precise nature remains elusive for cold stress-induced cardiovascular dysfunction, endothelin (ET-_A_) receptor, endoplasmic reticulum (ER) stress, transient receptor potential vanilloid, mitochondrial-related protein including NRFs and UCP-2, ROS, Nrf2-Keap1 signaling pathway, Bcl-2/Bax, and lipoprotein lipase (LPL) signaling may all play a pivotal role. For myocardial injury evoked by cold stress, more comprehensive and in-depth mechanisms are warranted to better define the potential therapeutic options for cold stress-associated cardiovascular diseases.

## Introduction

Sustained exposure to cold climate is one of the main risk factors that adversely affect human health. Numerous epidemiological studies, including Asia, Europe, and the America, have noted a tight correlation between cold climate and increased cardiovascular mortality (Keatinge, 1997; Mercer, 2003; Ogawa et al., 2007; Berko et al., 2014; Zhou et al., 2014). Moreover, cold weather also leads to an increase in the number of patients admitted to the emergency department (Baumgartner et al., 2008; Chen et al., 2019). Ample clinical and epidemiological studies have confirmed that cold weather is associated with increased adverse cardiovascular events (Keatinge, 1997; Stewart et al., 2002; Medina-Ramón et al., 2006; Cheng and Su, 2010; Ikäheimo, 2018). These findings indicate that cold stress can be considered as an independent risk factor for cardiovascular events. However, mechanisms responsible for adverse cardiac changes induced by cold stress remain unclear. Over the past decades, cold stress was noted to trigger a series of pathophysiological changes in cardiac function. These studies have revealed possible molecular pathways associated with cold stress-induced cardiovascular events and potential treatment options for cold stress-induced myocardial injury.

In this review, we summarized pathophysiological changes in myocardial injury induced by cold stress. We will further summarize specific molecular pathways that play an important role in cold stress-induced cardiovascular anomalies and provide a more comprehensive understanding of myocardial injury induced by cold stress.

## Myocardial Pathophysiological Changes Induced by Cold Stress

### Cardiac Structure and Function

Animal experiments reveal that cold exposure does not cause overt changes in body weight or size (Bello Roufai et al., 2007; Zhang et al., 2012b; Jiang et al., 2013; Matsubara et al., 2016). Nonetheless, cold exposure is associated with significant alterations in heart weight (Fregly et al., 1989; Bello Roufai et al., 2007; Cheng and Hauton, 2008; Yin et al., 2015; Matsubara et al., 2016; Liang et al., 2017), left ventricular (LV) wall thickness (Bello Roufai et al., 2007; Templeman et al., 2010; Zhang et al., 2012b; Liang et al., 2017), LV end-diastolic (LV EDD; Cheng and Hauton, 2008; Zhang et al., 2012b; Jiang et al., 2013), and LV mass (Zhang et al., 2012b). A transverse mice heart section showed that compared with the control group, cold stress significantly thickened the LV wall. In another study, histological staining sections revealed increased cardiomyocyte area following cold exposure, which was consistent with increased LV mass, wall thickness, EDD, and heart weight (Zhang et al., 2012b; Yin et al., 2015; Nagasawa et al., 2016; Liang et al., 2017). These changes indicate that cold stress leads to changes in cardiac structure and cardiac hypertrophy. However, scientists have observed that cold exposure does not affect LV wall thickness, LV mass (Zhang et al., 2012a; Jiang et al., 2013), and LV EDD (Zhang et al., 2012a). These differences may be related to the extent and duration of cold stress in different experiments. Evaluation of cardiac function using echocardiography has indicated that cold stress decreases in ejection fraction and shortening fraction of mouse hearts, leading to dampened LV function (Zhang et al., 2012b; Jiang et al., 2013; Yin et al., 2015; Cong et al., 2018).

### Fibrosis and Cardiomyocyte Injury

Masson trichrome staining revealed overt fibrosis in cold stress-exposed mouse heart, demonstrating that cold exposure prompted overt interstitial fibrosis in the heart (Zhang et al., 2012a,b; Yin et al., 2015; Nagasawa et al., 2016; Liang et al., 2017; Cong et al., 2018). Myocardial hypertrophy is often characterized by myocardial ultrastructural damage. Further observation of murine cardiomyocytes using transmission electron microscope depicted that cold stress led to sarcomere loss, myofibril disarray, and myofilament breakage. Besides, a high number of altered mitochondria was observed with different degrees of alterations such as swelling, vacuolation, autophagy, and a decrease in the number as well as disruption of cristae with formation of large vesicles, indicating significant degenerative changes (Bombig et al., 2003; Daud et al., 2009; Yin et al., 2015; Liang et al., 2017; Wang Z. et al., 2020). Vacuolar degeneration, eosinophilic degeneration, increased macrophage infiltration (Nagasawa et al., 2016; Cong et al., 2018), and nuclear shrinkage has also been reported to occur in cardiomyocytes (Meneghini et al., 2009).

Experimental observations have confirmed that cold stress induces cardiomyocyte injury, overt interstitial fibrosis, mitochondrial damage, and inflammation of the heart.

## Mechanism of Myocardial Injury Induced by Cold Stress

### Oxidative Stress

Changes in oxidative stress products, antioxidant defense enzymes, and non-enzymatic antioxidants in myocardium have been observed in nearly all cold stress studies. Research has revealed that cold stress may cause an increase in the metabolic rate and increased production of reactive oxygen species (ROS), such as hydrogen peroxide (H_2_O_2_), hydroxyl radicals (HO), and superoxide anion radicals (O_2_^–^⋅), which cause lipid peroxidation (Selman et al., 2000). At the same time, the body produces enzymatic Cu, Zn-superoxide dismutase (SOD-1), catalase (CAT), selenium-dependent glutathione peroxidase (Se-GSH-Px), and non-enzymatic antioxidants, such as reduced glutathione (GSH) to neutralize excessive ROS. Therefore, the damage caused by cold to the myocardium may be caused by an imbalance between oxidants and antioxidants in the body (Sahin and Gümüşlü, 2004).

Experimental findings have shown that sustained cold exposure promotes ROS production (Zhang et al., 2012a,b; Jain et al., 2013; Jiang et al., 2013; Wang et al., 2013), carbonyl formation, and generation of superoxide (O_2_^–^; Jiang et al., 2013) in the myocardium. 3′-Nitrotyrosine (3′-NT) and 4-hydroxynonenal (4-HNE) represent two markers for myocardial oxidative stress (Asselin et al., 2013). These markers are elevated with sustained cold exposure (Cong et al., 2018). More studies have found that cold exposure combined with hypoxia causes a significant increase in the level of malondialdehyde (MDA), a marker of oxidative stress (Jain et al., 2013). Cold stress has also been reported to cause an increase in the production of superoxide in myocardial tissue slices, and the activity of NADPH oxidase in LV tissue homogenate increases significantly (Nagasawa et al., 2016). Furthermore, other studies have found that cold combined with hypoxia causes a double increase in free radicals in the myocardium. The hypoxia-inducible factor (HIF-1α) is considered a key marker of cell response to hypoxia and plays a vital regulatory role in facilitating oxygen transport in tissues (Semenza, 2004). When there is cold climate and poor oxygenation in the environment, hypoxia-inducible factor expression is overtly upregulated (Zhang et al., 2012a; Jain et al., 2013). These results indicate that collectively, acute hypothermia and hypoxia significantly intensifies free radical-mediated physiological response in the myocardium. Some studies have revealed the role of oxidative stress in myocardial injury caused by cold stress through antioxidants. Metallothionein, a low molecular weight heavy metal-chelating antioxidant (Yang et al., 2019), significantly improves ROS overproduction, myocardial fibrosis, and myocardial contraction dysfunction caused by cold stress (Zhang et al., 2012a).

**FIGURE 1:**
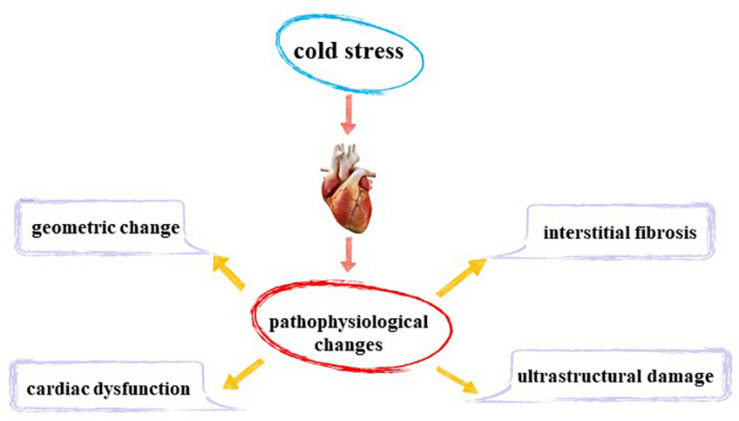
Myocardial pathophysiological changes induced by cold stress.

Besides pro-oxidation products, levels and activities of various antioxidant enzymes are also altered in response to cold stress. Experimental studies have shown that sustained cold exposure decreases levels of GSH and GSH/GSSG ratio not only in the myocardium (Zhang et al., 2012a; Jiang et al., 2013) but also in nearly all tissues (Kaushik and Kaur, 2003; Sahin and Gümüşlü, 2004). Western blotting analysis also demonstrated that levels of superoxide dismutase SOD-1 and Mn-superoxide dismutase (SOD-2) in mice decreases after 2 weeks of cold exposure. The expression of SOD-1 and SOD-2 mRNA in LV myocardium, detected by real-time RT-PCR, also indicated similar changes as observed with protein expression (Cong et al., 2018). In terms of antioxidant enzyme activity, SOD-1 and CAT activities have been shown to significantly decrease in the heart due to cold stress (Kaushik and Kaur, 2003; Sahin and Gümüşlü, 2004). The reason for this phenomenon may be that SOD-1 catalyzes the production of a large amount of H_2_O_2_, resulting in product inhibition. However, other studies noted an upregulated CAT and Se-GSH-Px activities in the myocardium following cold stress (Sahin and Gümüşlü, 2004). These results revealed that cold stress provokes an imbalance between oxidative stress and antioxidant defense, leading to myocardial injury. An independent study conducted in humans revealed a significant increase in the activities of Se-GSH-px, GSH, and CAT in erythrocytes from short track skaters exposed to prolonged cold environment, which may represent a protective mechanism against ROS damage (Hong et al., 2008; Cong et al., 2018). Therefore, myocardial injury induced by cold stress may be caused by an imbalance between oxidation and antioxidation and thus elimination by antioxidants.

To explore specific pathways and mechanisms of oxidative stress in myocardial injury induced by cold stress, ample experimental studies have been conducted. Nrf2 is a cell sensor protein with an important role in antioxidation, while Keap1 initiates its autophagy degradation (Tonelli et al., 2018). Cold stress has been reported to reduce protein expression of Nrf2 and keap1. Similar trends were noted in mRNA expression of these two genes (Cong et al., 2018). Oxidative stress changes amino acid residues in Keap1 structure, resulting in dysfunction (Cui et al., 2015; Barančík et al., 2016). These findings imply that myocardial oxidative stress injury provoked by cold stress develops likely through the Nrf2-Keap1 signaling pathway.

### Autophagy

Autophagy plays an important role in many physiological and pathological processes, including regulating the degradation of macromolecular substances, organelles, and nutrients, which are positively correlated with cold exposure (Williams et al., 2010; Nemchenko et al., 2011). Autophagy plays a vital role in the quality control of organelles and intracellular proteins, and autophagy products, such as amino acids and fatty acids, provide raw materials for the synthesis of adenosine 5′-triphosphate (ATP), proteins, and organelles. At the same time, autophagy also plays an important role in regulating intracellular secretion and transportation and was initially (Kroemer, 2015) considered a nonspecific degradation process, although specific autophagy degradation was recently noted. Mitophagy is a specific autophagy that clears damaged mitochondria and plays an important role in mitochondrial quality control (Shirakabe et al., 2016). Mitochondrial quality control of cardiomyocytes is very critical for heart function because mitochondria of cardiomyocytes produce a large amount of ATP daily to maintain the heart’s blood-pumping function. In a normal physiological state, mitochondria function is consistent with energy demand of the heart, to maintain normal heart function (Saito and Sadoshima, 2015; Nirwane and Majumdar, 2018). Therefore, cardiac autophagy is one of the important ways of maintaining cardiac homeostasis, and in some cases, autophagy activation can protect the heart. However, when the external injury persists, excessive autophagy is also considered to be related to the pathological changes of the heart (Delbridge et al., 2017). Autophagy results include adaptation, apoptosis, and necrosis. When autophagy is activated by various stresses, adaptation occurs in most cases, which limits cell dysfunction and causes cell death; however, in some cases, it results in apoptosis and necrosis (Sciarretta et al., 2018). Autophagy can resist heart injury under various pathological conditions by eliminating misfolded proteins, damaged organelles, dysfunctional mitochondria, and inhibiting oxidative stress, and can alleviate systolic dysfunction and cardiac structural remodeling during hemodynamic overload, to maintain the normal structure and function of the heart (Nakai et al., 2007; Maejima et al., 2013; Ikeda et al., 2015; Yin et al., 2020).

Excessive accumulation of PTEN-induced putative kinase 1 (PINK1) protein phosphorylates Parkinson’s disease protein 2 (Parkin) protein and initiates the formation of mitochondrial autophagy (Yamano et al., 2016). Compared with the control group, proteins related to mitochondrial autophagy, PINK1, and phosphorylation level of Parkin were reported to be significantly increased during cold exposure (Wang Z. et al., 2020). Studies have found that cold exposure significantly upregulates the levels of essential protein markers of autophagy including LC3B-II, LC3B-II/LC3B-I ratio, and beclin-1, Atg7, and ULK1 Ser777 phosphorylation, indicating facilitated autophagy following cold exposure. A large number of LC3+ puncta have been observed in the myocardial slices of murine after cold exposure by fluorescence immunohistochemistry. Western blot and fluorescence immunohistochemistry showed that inhibition of autophagy significantly alleviated an increase in LC3+ puncta induced by cold exposure. Furthermore, inhibition of autophagy can significantly reduce the abnormal mechanical function of myocardial cells caused by cold compared with normal conditions (Jiang et al., 2013). These results indicate that autophagy plays a significant role in myocardial contraction induced by cold stress. The BH3 domain of Bcl-2 binds to beclin-1, and the conformation of beclin-1 may be altered by phosphorylation in the BH3 domain, resulting in the release of beclin-1 from the complex and induction of autophagy (Qi et al., 2011). Immunoprecipitation results found that the amount of Bcl-2 that immunoprecipitated with beclin-1 was significantly decreased in mice exposed to low temperatures. This indicates that cold stress can trigger the dissociation of between Bcl-2 and beclin-1, leading to autophagy (Jiang et al., 2013). It was reported earlier that cold exposure significantly augmented pressure overload-induced phosphorylation of AMP-dependent protein kinase (AMPK; Lu and Xu, 2013), which serves as a positive regulator of autophagy (Shaw, 2009). All these findings showed a possible role of autophagy induction in cold stress-induced changes in the myocardial. Akt and mammalian target of rapamycin (mTOR) are two proteins that inhibit autophagy. Assessment of signaling mechanisms involved in autophagy regulation revealed that cold exposure-induced autophagy is related to inhibition of the phosphorylation of the two proteins (Williams et al., 2010; Nemchenko et al., 2011; Xu and Ren, 2012). Autophagy is strictly regulated by upstream mediators, and both Atg (autophagy-related gene) family and mTOR kinase can inhibit autophagy (Ren et al., 2008; Williams et al., 2010). The phosphorylation level of Akt and mTOR is also reported to decrease after cold stress exposure. mTOR is the main inhibitor of autophagy and plays a key role in the regulation of autophagy, while Akt is the most important upstream activator of mTOR autophagy (Jiang et al., 2013). Therefore, reduced phosphorylation of Akt and mTOR further reveals their signaling role in cold stress-induced regulation of myocardial autophagy.

**FIGURE 2:**
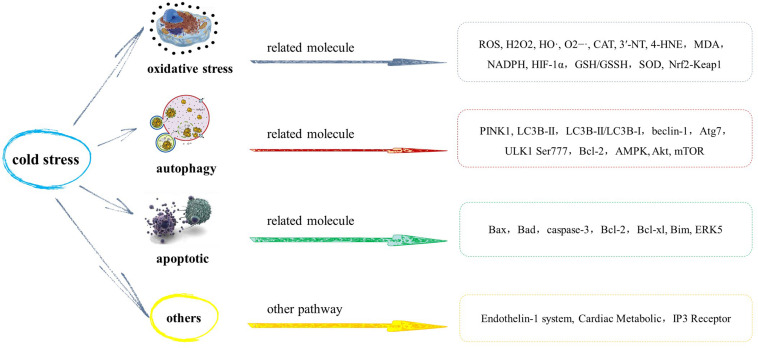
Mechanism of myocardial injury induced by cold stress.

### Apoptotic

Through staining to detect apoptosis rate of cardiomyocytes *in vitro*, cold stress was found to significantly increase the apoptosis rate (L’Ecuyer et al., 2012; Zhang et al., 2012a,b; Wang et al., 2013; Yin et al., 2015; Liang et al., 2017). Apoptosis-related proteins include proapoptotic proteins and antiapoptotic proteins. The proapoptotic proteins include Bax, Bad, and caspase-3, while Bcl-2 belongs to antiapoptotic protein (Delbridge et al., 2016; Pereira et al., 2018). The expression of Bax and Bad was reported to be increased, and the expression of Bcl-2 decreased after 2 weeks of cold exposure (Yin et al., 2015; Liang et al., 2017; Cong et al., 2018; Wang Z. et al., 2020). The mRNA expression in Bax and Bad was significantly elevated in the cold exposed groups, while the expression of mRNA in Bcl-2 decreased (Cong et al., 2018). The protein and gene detection results are consistent, and cold exposure promotes cardiomyocyte apoptosis by decreasing Bcl-2/Bax levels. The production and activity of caspase-3 were also found to be significantly elevated in cardiomyocytes in cold-stressed mice (Zhang et al., 2012b; Yin et al., 2015).

However, some studies have differing results, although cold exposure is reported to lead to an increase in apoptosis and an increase in the expression of the proapoptotic protein Bax in the cold-exposed group. However, there is no significant change in Bcl-xl; hence, Bcl-xl dose not participate in cold-induced myocardial apoptosis (Lai et al., 2009; Zhang et al., 2012a). Bcl-xl is a member of the antiapoptotic protein Bcl-2 family, a transmembrane molecule in the mitochondria (Lai et al., 2009). Bim, which is widely expressed in different organs, is an important proapoptotic protein and belongs to the Bcl-2 family (Czabotar et al., 2013). Hypothermal stimulation induces increased Bim expression in cardiomyocytes and extracellular signal-regulated kinase 5 (ERK5) knockdown, which can further promote increased Bim expression. Further studies have reported that knocking down Bim can attenuate hypothermia stimulation-induced apoptosis, while knocking down ERK5 can increase hypothermia stimulation-induced apoptosis. These results suggest that the expression of proapoptotic protein Bim in the myocardium may be regulated by ERK5. ERK5 has an antiapoptotic effect and is the upstream regulator of Bim (Wang et al., 2013).

### Others

In addition to the pathways discussed above, some other pathways also play a role in myocardial injury caused by cold stress.

### Endothelin-1 System

Endothelin (ET)-1 is a vasoconstrictor peptide secreted by vascular endothelial cells, which play an important role in maintaining vascular tension. It is one of the most effective endogenous vasoconstrictors and an important participant in coronary atherosclerotic heart disease and myocardial infarction (Skovsted et al., 2017). It is reported that ET-1 is a local hormone whose secretion varies according to changes in the surrounding environment. Cold exposure increases the secretion of ET in plasma and increase in ET-1 in the heart tissue (Chen and Sun, 2006; Barton and Yanagisawa, 2008; Wang D. et al., 2020). ET-1 plays an important role in the regulation of cardiac growth, myocardial contractility, and hemodynamics, and also leads to pathological myocardial hypertrophy by activating phosphoinositide 3-kinases (PI3Ks)/Akt pathway related to myocardial contractility and mitogen-activated protein kinase (MAPK)/extracellular signal-regulated kinases (ERK1/2) pathway related to cell hypertrophy (Drawnel et al., 2013; Takano et al., 2020). The effect of ET-1 on myocardial contractility and arrhythmia causes pathological remodeling of the heart (Drawnel et al., 2013). The knockout of the ET-1 receptor significantly counteracts cardiac hypertrophy and contractile dysfunction induced by cold. It was noted that knockout of ET-1 receptor causes a decrease in temperature-sensor protein in conjunction with dampened mitochondrial function (Zhang et al., 2012b).

The temperature sensor protein transient receptor potential vanilloid (TRPV1) receptor is an important protein that maintains myocardial contraction. TRPV1 acts as a major cold sensor to prevent nociceptive cold stimulation (Karashima et al., 2009). Cold stress causes changes in the structure and function of the heart, such as LV hypertrophy and decreased contractility, and TRPV1 agonists can significantly alleviate these adverse changes. TRPV1 antagonist stimulates a decrease in myocardial contractile function induced by cold stress. Consistent with this finding, knockout ET-1 receptors reverse downregulation of TRPV1, thereby improving cardiac hypertrophy and dysfunction induced by cold (Zhang et al., 2012b). These results show that TRPV1 plays a significant role in the changes of heart injury caused by cold stress.

Glycogen synthase kinase-3β (GSK3β) is a signal molecule that regulates the geometry of the myocardium, the integrity of the mitochondrial structure, and the survival of cardiomyocytes (Cheng et al., 2011). PGC1α and UCP2 are proteins responsible for mitochondrial oxidative phosphorylation and biogenesis (Finck and Kelly, 2006; Rey et al., 2010). Cold is reported to upregulate UCP2, downregulate PGC1α, and enhance GSK3β phosphorylation. In addition, knockout of the ET-1 receptor eliminates these protein changes caused by cold, and besides, knockout of the ET-1 receptor significantly alleviates cold-induced damage to heart structure and function (Zhang et al., 2012b).

Cold increases the level of ET-1 in plasma and myocardial tissue, and ET-1 plays an important role in the regulation of cardiac physiological and pathological function. The ET system also plays an important role in the governance of myocardial homeostasis, which may be involved in cold stress-provoked cardiovascular defect.

### Cardiac Metabolism

The heart is an organ necessitating high-energy consumption, in particular, continuous energy demand is continuous. This energy is derived from nutrients in plasma (Lopaschuk et al., 2010). Short-chain hydroxy acyl-coenzyme A dehydrogenase (SCHAD) is a key transcriptional regulator of fatty acid catabolism enzymes and plays a regulatory role in cardiac metabolic remodeling (Brown et al., 1995; Aoyama et al., 1998). Chronic cold stress has been found to reduce the activity of SCHAD in the left ventricle by one third (Templeman et al., 2010). Previous studies have found that chronic cold stress does not affect oxygen supply to the heart, since brown adipose tissue has a higher demand for fatty acids, and this changes the amount of energy provided to the heart (Kayar and Banchero, 1985; Hauton et al., 2009). Therefore, myocardial hypertrophy caused by cold stress causes a decrease in the heart fatty acid oxidation ability, which further leads to a series of subsequent myocardial injuries (Templeman et al., 2010).

Chronic cold stress aggravates abnormal glucose and lipid metabolism in metabolic syndrome model animals. Glucocorticoid receptor (GR) antagonists alleviate metabolic abnormalities caused by cold stress (Nagasawa et al., 2016). GR widely exists in the myocardium and blood vessel walls (Walker, 2007), and glucocorticoids have a certain immunosuppressive effect. This indicates that the glucocorticoid-GR signaling pathway is involved in the heart injury of metabolic syndrome mice induced by cold stress. However, there is no available data on GR pathway changes in the heart of normal mice after cold stress; therefore, it is impossible to infer whether the heart of people with metabolic diseases is more vulnerable to damage by the cold.

### IP3 Receptor

Inositol 1,4,5-trisphosphate (IP3) is the second messenger involved in G protein-coupled receptor-mediated signal transduction. Extracellular signals, such as growth factors and neurotransmitters, stimulate the formation of IP3 by activating G protein-coupled receptors. The IP3 functions by opening the Ca^2+^ channel and binding to the IP3 receptor on the sarcoplasmic reticulum (SR) and nuclear envelope, resulting in release of Ca^2+^ from the intracellular pool (Berridge, 2016). Under normal physiological conditions, IP3 receptors respond to the needs of the cells under precise regulation *in vivo* and closely control a variety of Ca-dependent physiological processes. The IP3/Ca^2+^ signaling pathway regulates many cellular physiological processes and plays a key role in the development of many diseases (Berridge, 2016; Prole and Taylor, 2019; Ivanova et al., 2020). There are three types of IP3 receptor isoforms, IP3R1, IP3R2, and IP3R3. Their distribution in the heart is significantly different, and they have different physiological characteristics and functions. IP3R1 is mainly distributed in the cardiac ganglia, IP3R2 is mainly distributed in cardiomyocytes, and IP3R3 is distributed in the atrial cell junction and plays an important role in atrial excitation–contraction coupling. The action of ET-1 on IP3R3 enhances the release of Ca^2+^ and spreads to the whole cell, thus enhancing the contractile force (García et al., 2004; Slavikova et al., 2006; Berridge, 2016). The regulation of Ca^2+^ release by IP3 plays an important role in the development of myocardial hypertrophy (Nakayama et al., 2010). In cardiomyocytes, an increase in the expression of IP3R3 is stimulated by various factors, and the ectopic calcium released induces the expression of the hypertrophy gene and promotes arrhythmia (Drawnel et al., 2012; Berridge, 2016). IP3-mediated plasma membrane calcium signal transduction regulates the contraction of atrial myocytes and participates in the arrhythmogenic effect of ET-1, and this may be the ionic basis for ET-1 stimulation of cardiomyocytes to enhance automaticity. Atrial arrhythmia is the most common arrhythmia caused by abnormal IP3/Ca^2+^ signal pathway. Similarly, IP3 plays a similar role in the sinoatrial node (Drawnel et al., 2013; Hohendanner et al., 2015). IP3R2 integrates the contractile Ca^2+^ signal to generate the nuclear Ca^2+^ signal, which activates the transcriptional events that cause hypertrophy (Berridge, 2016). Previous studies found that after 28 days of cold exposure, the mRNA and protein level of IP3R1 increased significantly, the mRNA level of IP3R2 significantly increased, but its protein expression showed an increasing trend, but with no significant difference (Krizanova et al., 2008). Besides, the expression of the IP3R1 mRNA gene in the left atrium was significantly upregulated after cold exposure for 7 days (Krizanova et al., 2005). These results are consistent with the finding that Ca^2+^ of mice cardiomyocytes increased significantly after cold exposure (Zhang et al., 2012a). These results suggest that the IP3/Ca^2+^ signaling pathway plays a role in heart injury caused by cold exposure. Cold stress upregulates the IP3 receptor in cardiomyocytes and causes the release of intracellular Ca^2+^, leading to arrhythmia and myocardial hypertrophy through the IP3/Ca^2+^ signaling pathway, and finally leads to cardiac geometric remodeling.

## Summary

Existing experimental data on myocardial injury caused by cold stress showed that cold stress can lead to oxidative stress injury, promote autophagy and apoptosis. Cardiomyocytes are vigorous aerobic cells that produce a series of ROS, including O_2_^–^, H_2_O_2_, HO_2_, and OH, etc. At an appropriate concentration, ROS maintains the normal physiological function of cells, including activation of transcription factors and promotion of normal differentiation and proliferation of cells. However, when the concentration of ROS is too high, it causes oxidative stress, which leads to cell dysfunction and apoptosis (Wang et al., 2013). When cardiomyocytes are in a hypothermal environment, various subcellular changes take place in the cells. Hypothermia can cause damage to mitochondrial biological function, and the degree of damage is proportional to an increase in ROS in the myocardium (Camara et al., 2004). Mitochondria and apoptosis are closely related, since a decrease in mitochondrial transmembrane potential (ΔΨm) induces apoptosis, and this process is irreversible. Further, this leads to a series of subsequent pathophysiological processes. For example, it triggers cell apoptosis by uncoupling the respiratory chain and oxidative phosphorylation to stop ATP synthesis, or disrupt the mitochondrial membrane permeability, and open the mitochondrial permeability transition pore (Wang et al., 2013). Previous studies have found that cardiomyocyte apoptosis induced by pressure overload can lead to cardiac hypertrophy, and apoptosis may be involved in the pathological process of cardiac remodeling (Teiger et al., 1996). Myocardial fiber is ubiquitous in many cardiovascular diseases, which results in an imbalance between collagen synthesis and metabolism. It was noted that cold stress causes myocardial fibrosis. Autophagy is an important catabolic system, which plays an essential role in maintaining cellular physiological function. Studies have shown that autophagy plays an important role in myocardial fibrosis (Shimizu and Minamino, 2016; Lu et al., 2018). In addition to the aforementioned pathways, the ET system also plays an important role in myocardial injury caused by cold stress. ET-1 is one of the most important endogenous vasoconstrictors, which regulates the cardiovascular system and myocardial contractility, affecting cardiac remodeling, and so on. Cold stress causes an increase in ET-1 secretion, which leads to myocardial hypertrophy, cardiac systolic dysfunction, and so on. GSK3 proteins related to the regulation of myocardial geometry and TRPV1 proteins related to cold receptors are involved in this process. Metabolic disorders can lead to oxidative stress and damage to multiple organs throughout the body (Wu and Ren, 2006; Li et al., 2007). Cardiac metabolic disorders and IP3-mediated Ca^2+^ disorders also play an important role in myocardial damage, as well as in cardiac geometric remodeling and ultimately cardiac dysfunction (Xu et al., 2020). In conclusion, the myocardial injury caused by cold is a multipathway and multimolecular process, and a variety of measures can be taken to prevent and treat the myocardial injury caused by cold stress.

## Author Contributions

XK and HL searched the articles and retrieved the information from the included studies. XK drafted this article. XH and YS put forward some amendments to the article. WG supervised the whole process of this review. All authors contributed to the article and approved the submitted version.

## Conflict of Interest

The authors declare that the research was conducted in the absence of any commercial or financial relationships that could be construed as a potential conflict of interest.
